# Methylnaltrexone’s Effect on Cholestasis in Trauma Patients

**DOI:** 10.7759/cureus.69750

**Published:** 2024-09-19

**Authors:** Andrew McCague, Ellie G Wallace, Rebecca Shaneck, Jacky Kamel, Hal Piwonka

**Affiliations:** 1 Trauma and Acute Care Surgery, Desert Regional Medical Center, Palm Springs, USA; 2 General Surgery, Trauma Surgery, Western University of Health Sciences, Pomona, USA; 3 Pharmacy, Desert Regional Medical Center, Palm Springs, USA

**Keywords:** acute acalculous cholecystitis (aac), cholestasis, methylnaltrexone, opiate use, trauma

## Abstract

Introduction

Cholestasis in hospitalized patients receiving opiates has the potential to have devastating outcomes including acalculous cholecystitis, sepsis, or even death. In this study, we evaluate the outcomes of trauma patients treated with methylnaltrexone.

Methods

We conducted the study at Desert Regional Medical Center, a level 1 trauma center in Palm Springs, California. Our electronic medical record was queried for all trauma patients between January 1, 2018 and January 1, 2024.

Patients were excluded if they were under 18 years old or had a documented history of cirrhosis, congestive heart failure, or cardiac arrest during their admission. Those not excluded were divided into a treatment group including those who received at least one dose of methylnaltrexone and those who never received a dose of methylnaltrexone. A control group was created matching by randomly selecting one patient from the non-treatment group for each patient in the treatment group. The control and treatment groups were then analyzed using SPSS (IBM Corp., Armonk, NY). Demographic data was compared using student t-test between the two groups including age, sex, length of stay, ICU length of stay, ventilator days, and mortality. A p-value is reported as a test statistic. The incidence of cholestasis was measured by either laboratory evidence of hyperbilirubinemia, radiologic evidence of cholecystitis, diagnosis code of cholecystitis, or procedural intervention for cholecystitis. The two groups were compared for incidence of cholestasis using student t-test. Results are reported below.

Results

A total of 9894 patients were evaluated for inclusion in the study. Of those patients, 1292 patients met the exclusion criteria, and 8602 patients were analyzed for administration of the study drug. Fifty-six patients received at least one dose of methylnaltrexone. The remaining 8597 patients did not receive the treatment. Fifty-six patients were selected randomly for the control group.

The median age between the treatment and control groups did not show statistically significant differences. Sex was also not statistically different between the two groups with 38 males and 18 females in the treatment group as compared to 39 males and 17 females in the control group with a p-value of 0.86. The median hospital length of stay was longer in the treatment group at 13 days compared to only one day in the control group which was statistically different with a p-value of <.001. ICU length of stay was also found to be statistically different between the treatment and control groups with 4 and 0 days respectively and a p-value of <.001. Mortality between the two groups was also higher in the treatment group with five patients in the treatment group not surviving to discharge as compared to one patient in the control group (p-value = .044). Both groups had one patient who met the criteria for cholestasis representing an overall incidence of 1.8% for each group.

Conclusion

Our data suggests that patients who received the medication were overall sicker with longer hospital stays, increased mortality, and more likely to have required surgery. We did not show a significant difference in incident of cholestasis or acalculous cholecystitis between the two groups but suggest that a larger study is warranted to study a causal relationship.

## Introduction

Opioid-induced cholestasis and ultimately acalculous cholecystitis stands as a formidable clinical challenge, characterized by the inflammation of the gallbladder often precipitated by prolonged opioid use [1,2]. Although opioids are instrumental in preventing pain crises in critically ill patients, they can cause significant gastrointestinal side effects, including opioid-induced constipation (OIC), increased biliary pressure, and sphincter of Oddi spasm [3,4]. Cholestasis caused by opioid side effects can result in acalculous cholecystitis [5,6]. Acalculous cholecystitis is the inflammation of the gallbladder without the presence of a stone, which is commonly complicated by gangrene or perforation, and can increase mortality and lengthened ICU stays [7,8].

Patients with prolonged hospital stay have been shown to have an increased incidence of cholestasis. Among the arsenal of pharmacological interventions, methylnaltrexone has emerged as a promising candidate for reversing opioid-induced cholestasis [9, 10]. Methylnaltrexone, a peripherally acting mu-opioid receptor antagonist, offers a unique mechanism of action that selectively targets opioid-induced gastrointestinal dysmotility while preserving central analgesic effects [11,12]. Methylnaltrexone has been used for the management of cholestasis related to cholangitis due to its reported effect on helping relax the sphincter of Oddi. This distinctive pharmacological profile positions methylnaltrexone as a potential game-changer in the management of opioid-induced cholestasis that has the potential to proceed to acalculous cholecystitis [13,14].

By interrogating the therapeutic potential of methylnaltrexone in reversing opioid-induced cholestasis, this study aims to pave the way for evidence-based interventions that alleviate suffering, improve patient outcomes, and mitigate the burden of opioid-related complications on healthcare systems worldwide [15-18].

## Materials and methods

We conducted the study at Desert Regional Medical Center, a level 1 trauma center in Palm Springs, California. Approval was obtained from MetroWest Medical Center Institutional Review Board prior to the initiation of this study. Our electronic medical record was queried for all trauma patients between January 1, 2018 and January 1, 2024. A de-identified dataset was created including demographic data, trauma injury data, diagnosis codes, and dosages of methylnaltrexone received.

Patients were excluded if they were under 18 years old or had a documented history of cirrhosis, congestive heart failure, or cardiac arrest during their admission. Patients were identified who were found to have the diagnosis of cholestasis or acalculous cholecystitis based on laboratory evidence of elevated bilirubin, diagnosis code of cholecystitis, radiographic evidence of cholecystitis, or who underwent a procedure for cholecystitis. Those not excluded were divided into a treatment group including those who received at least one dose of methylnaltrexone and those who never received a dose of methylnaltrexone. A control group was created matching by randomly selecting one patient from the non-treatment group for each patient in the treatment group. The treatment and randomly created control groups were then compared and analyzed in SPSS (IBM Corp., Armonk, NY). Demographic data was compared using student t-test between the two groups including age, sex, length of stay, ICU length of stay, ventilator days, and mortality. A p-value is reported as a test statistic. The incidence of cholestasis was measured by either laboratory evidence of hyperbilirubinemia, radiologic evidence of cholecystitis, diagnosis code of cholecystitis, or procedural intervention for cholecystitis. The two groups were compared for incidence of cholestasis using student t-test. Results are reported below.

## Results

**Figure 1:**
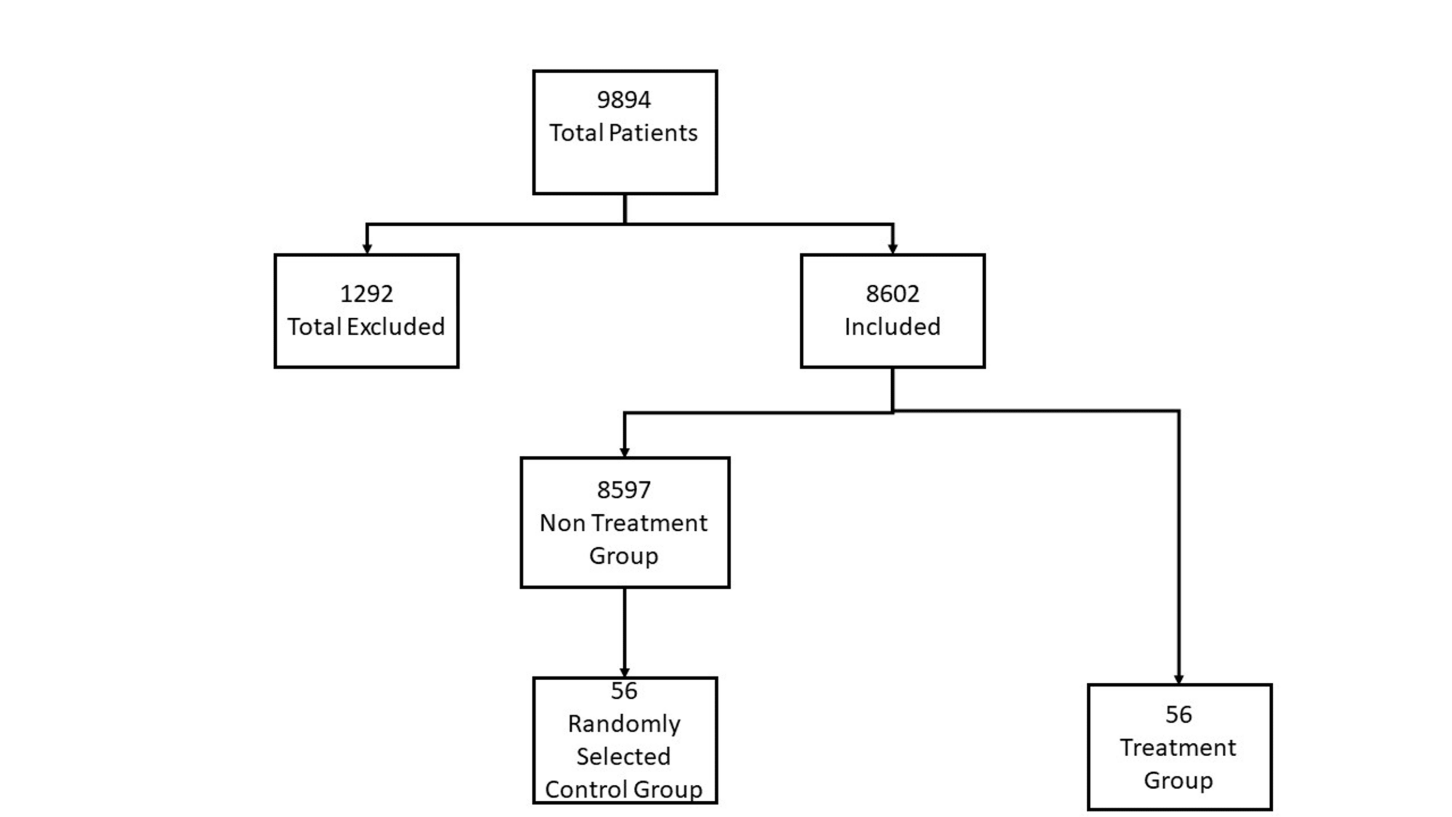
Breakdown of included and excluded patients with control and treatment groups.

A de-identified dataset was reviewed. A total of 9894 patients were found to have suffered traumatic injuries during the study period. Of those patients, 1292 patients met the exclusion criteria and were removed from the analysis. The remaining 8602 patients were analyzed for administration of the study drug. Fifty-six patients were found who had received at least one dose of methylnaltrexone. The remaining 8597 patients did not receive the treatment. Using a random number generator, a control group was created matching patients in the treatment group with randomly selected patients in the non-treatment group. Fifty-six patients were selected randomly for the control group. Figure 1 describes the breakdown of patients included and excluded.

The treatment group of 56 patients was compared to the randomly generated control group of 56 patients. Demographic data is presented in Table 1. The median age between the treatment and control groups did not show statistically significant differences. The median age in the control group was 60 and 52 in the control group with a p-value of 0.26. Sex was also not statistically different between the two groups with 38 males and 18 females in the treatment group as compared to 39 males and 17 females in the control group with a p-value of 0.86.

**Table 1:** Demographic data compared between treatment and control groups.

	Treatment Group	Control Group	p-value
N	56	56	
Median Age (St. Dev.)	60 (19)	52 (22)	0.26
Sex			0.86
Males	38	39	
Female	18	17	
Median Length of Stay (St. Dev.)	13 (20)	1 (8)	< .001>
Median ICU Length of Stay (St. Dev.)	4 (13)	0 (2)	< .001>
Required Surgery	44	22	< .001>
Mortality	5	1	0.044
Median Number of Doses (St. Dev.)	3.5 (7)	0 (0)	< .001>
Biliary Stasis	1	1	1

The median hospital length of stay was longer in the treatment group at 13 days compared to only one day in the control group which was statistically different with a p-value of <.001. ICU length of stay was also found to be statistically different between the treatment and control groups with 4 and 0 days respectively and a p-value of <.001. The number of patients requiring any type of surgery was also evaluated as these patients would be anticipated to have a higher opiate requirement for pain. There was a statistically significant difference in patients requiring surgery between the two groups with 44 patients in the treatment group and 22 patients in the control group with a p-value of <.001. Mortality between the two groups was also higher in the treatment group with five patients in the treatment group not surviving to discharge as compared to one patient in the control group (p-value = .044). The incidence of cholestasis as determined using either laboratory evidence of elevated bilirubin, diagnosis code of cholecystitis, radiographic evidence of cholecystitis, or need for a procedure for cholecystitis was not different between the two groups. Both groups had one patient who met the criteria for cholestasis representing an overall incidence of 1.8% for each group.

An additional analysis was performed evaluating hospital disposition between the two groups. Table 2 shows an increased rate of discharge to home for the control group whereas the treatment group had an increased rate of discharge to rehab, skilled nursing facility, or expired.

**Table 2:** Hospital disposition for study patients.

	Treatment	Control Group
N	56	56
Home	28	39
Rehab or Skilled Nursing Facility	20	11
Expired	5	1
Against Medical Advice	1	2
Law Enforcement	1	2
Other Acute Care Hospital	1	1

Overall analysis between the two groups did not show a difference in the occurrence of cholestasis or acalculous cholecystitis. The treatment group results suggested a trend toward a sicker group of patients with a longer hospital stay, longer ICU stay, increased mortality, and less patients going home at the end of their hospital stay.

## Discussion

Cholestasis in hospitalized patients receiving opiates has the potential to have devastating outcomes including acalculous cholecystitis, sepsis, or even death. Methylnaltrexone use is growing in popularity in hospitals over recent years. Its mechanism as a mu-opioid receptor antagonist allows for the selective targeting of gastrointestinal dysmotility. It has been reported that methylnaltrexone also has effects on the sphincter of Oddi and may directly have effects on cholestasis. In this study, we evaluate the outcomes of trauma patients treated with methylnaltrexone.

Cholestasis is well documented to have the potential to progress to acalculous cholecystitis in the ICU setting. Although rare, acalculous cholecystitis comprises between 2-15% of all cases of acute cholecystitis [19]. When compared to calculous cholecystitis, mortality has been reported to be almost double, 9.6% versus 4.3% respectively when comparing acalculous to calculous cholecystitis [20].

Risk factors for the development of acalculous cholecystitis are generally associated with patients with prolonged hospital stays, multiple co-morbidities, and need for surgical intervention. Increased incidence of acalculous cholecystitis has been seen in sepsis, trauma, surgery, burns, multiple transfusions, and severe debilitation [21]. Dehydration might also increase bile viscosity, with both parenteral nutrition and mechanical ventilation reported to increase the incidence of cholestasis [20]. Multiple doses of opiates in postoperative trauma patients may lead to prolonged sphincter of Oddi spasm and perhaps increase luminal pressure in the gallbladder [22]. Studies have also suggested that hormones such as estrogen and progesterone as well as some medications including oral contraceptives, thiazides, ceftriaxone, octreotide, erythromycin, ampicillin, sunitinib, sorafenib, and alemtuzumab inhibit contraction of the gallbladder smooth muscle which can lead to cholestasis [23]. Rarely, viruses and parasites such as Epstein-Barr virus, hepatitis A virus, hepatitis E virus, Clonorchis sinensis, giardia, and Echinococcus species, may invade the wall of the gallbladder or biliary epithelial cells leading to cholestasis [23].

Although the precise mechanism is unclear, commonly postulated theories of pathophysiology of acalculous cholecystitis include bile stasis with a change in chemical composition causing local gallbladder inflammation, systemic sepsis, and ischemia [23, 24]. In critically ill patients, acalculous cholecystitis results from gallbladder ischemia, which may be secondary to shock from hypovolemia or sepsis [19]. Ischemia-reperfusion injury plays a major role in the pathogenesis of acalculous cholecystitis because the gallbladder artery is a terminal artery [23]. Ischemia can lead to necrosis of the gallbladder wall and later perforation. Instability related to either trauma or surgery, hypotension, and vasoactive drugs may also potentiate a decrease in perfusion to ischemia. Cholestasis itself is thought to increase the pressure within the gallbladder lumen leading to decreased perfusion of the gallbladder wall and ultimately ischemia [23].

Abdominal pain or signs of infection are often the first clinical signs of acalculous cholecystitis as a complication of cholestasis. Leukocytosis is common and serum bilirubin, liver enzymes, and amylase may be increased even to the point of mild jaundice [20]. Right upper quadrant ultrasound is a simple investigation that can be performed at the bedside even in unstable patients [20]. Positive findings include gallbladder wall thickening, distention of the gallbladder, sludge within the lumen, and pericholecystic edema [20]. Cholescintigraphy using technetium labeled iminodiacetic acid is more sensitive than ultrasound. Non-visualization of the gallbladder despite hepatic uptake and evidence of isotope passing into the small intestines is taken as positive evidence of cholecystitis [20].

Early identification and treatment can improve outcomes and length of hospital stay. The most optimal treatment for acalculous cholecystitis is cholecystectomy [20]. For critically ill patients or those who are unfit for surgery, percutaneous cholecystostomy tube placement is another option [24]. Reports have suggested that prophylactic cholecystokinin might overcome biliary stasis and prevent the development of acalculous cholecystitis [20]. We hypothesize that mu-opioid receptor antagonists may reverse the effects of either exogenous or endogenous opiate effects on the sphincter of Oddi allowing relief of biliary stasis [20].

Methylnaltrexone was approved by the US Food and Drug Administration for the treatment of opioid-induced constipation [25]. Methylnaltrexone, a peripheral mu-opioid receptor antagonist, is a derivative of naltrexone [25]. Naltrexone crosses the blood-brain barrier, resulting in pain and opioid withdrawal and is commonly used for overdose [26]. N-methylation of naltrexone limits its ability to cross the blood-brain barrier, thus persevering central analgesic effects and reversing the effects of opiates in the periphery including the GI tract [22]. Methylnaltrexone belongs to a new drug class with selective antagonism of peripheral mu-opioid receptors and might help relieve opioid-induced constipation but maintain analgesia [26].

Thomas et al. showed that methylnaltrexone reversed the opiate-induced delay in both gastric emptying and oral-cecal transit time without affecting analgesia [26]. There was a rapid response with patients receiving the medication responding within four hours and half of the patients responding within 30 minutes [26]. Critically ill patients may have other causes of their constipation including decreased mobility, decreased oral intake or fasting, a low fiber diet, metabolic and endocrine imbalances, neurologic disorders, concomitant drug side effects, inadequate toileting arrangements, sedation, depression and advanced age all of which can have effects on motility [26]. Many of these conditions can also contribute to cholestasis.

Several publications have reported effects on cholestasis with the use of methylnaltrexone, particularly pruritis. Pruritis is a well-recognized side effect of opiate use [27]. It has been hypothesized that endogenous opioid production in patients with cholestasis increases opioid agonists in the brain leading to a sensation of pruritis [27]. A case reported by Zhang et al. in 2013 described a patient who had metastatic non-small cell lung cancer and who had presumed paraneoplastic gastrointestinal dysmotility with gastroparesis and diffuse colonic dilation. The patient failed intravenous immunoglobulin therapy but improved with methylnaltrexone. The use highlighted non-obstructive biliary dilation as a rare manifestation of paraneoplastic gastrointestinal dysmotility [28]. Thus, the presence of colonic dilation and bile duct dilation was suggestive of opioid effects on the gastrointestinal tract. As the sphincter of Oddi has a high density of mu-opioid receptors, the non-obstructed dilation of the bile duct suggests a dysfunction of the sphincter. Here we hypothesize that the previously observed improvement of pruritis may actually be by improvement of cholestasis by methylnaltrexone’s effects on the sphincter of Oddi rather than the endogenous opiate effects alone.

Our study above looks at the effects of methylnaltrexone on cholestasis in trauma patients at our level I trauma center. Our treatment group was found to have overall longer length of stays and with presumed sicker patients. This suggests that methylnaltrexone is being used selectively in these patients rather than broadly across the trauma patient population. Each patient within the study groups had complex traumatic injuries and prolonged hospital stays with complicated courses.

In our treatment group, a 20-year-old male was admitted after a motor vehicle collision and spent 73 days in the hospital including 17 days in the ICU. His injuries include a facial fracture, splenic laceration, small bowel and colon injuries, pulmonary contusions, pneumothorax, traumatic pancreatitis, and spinal fractures. He underwent multiple surgeries including splenectomy, colectomy with ostomy, small bowel resection, open reduction internal fixation of facial fractures, and percutaneous cholecystostomy tube placement. His hospital course was complicated by cholecystitis, pulmonary embolism, and aspiration pneumonia. He was ultimately discharged home.

Our control group included a 31-year-old female who arrived at the emergency department after a motorcycle collision and was discharged home after 52 days in the hospital including 11 days in the ICU. She suffered a small bowel perforation, left ankle fracture, and spinal fractures. She required multiple surgeries including ostomy for a delayed bowel perforation. Her hospital course was complicated by cholecystitis, sepsis, pneumonia, DVT, ileus, and abdominal abscesses.

In our study, we did not show a statistically significant difference in incidence of cholestasis between the two groups. Given the small sample size and limited retrospective de-identified dataset, we cannot make direct conclusions as to the effects of methylnaltrexone on cholestasis or the incidence of acalculous cholecystitis within this patient population. We did see a trend among the groups of sicker patients with longer ICU and hospital stays as well as increased mortalities among those patients who received methylnaltrexone. We can hypothesize that providers caring for these patients had increased concern for opiate included constipation potentially related to the increased pain from surgery or trauma burden. A protocol is being considered to standardize the usage of methylnaltrexone at our institution.

This study is limited by the small sample size and use of a de-identified dataset. Cholestasis and acalculous cholecystitis, being rare in this population limited our sample size. A retrospectively created randomly assigned control group was used to improve the study design. The random generation of a control group helps remove sample bias. Diagnoses were based on ICD-10 codes and not independently validated from laboratory or radiology data. As a retrospective study, we are limited by dataset completeness. Our study also does not evaluate opiate doses or usage in the patients. As a significant risk factor for cholestasis and acalculous cholecystitis, future studies will need to evaluate the opiate exposure for each patient.

This study serves as an initial step in evaluating the effects of methylnaltrexone in a trauma population. A larger prospective study comparing treatment and placebo groups would allow more definitive conclusions to be drawn.

Cholestasis and its progression to acalculous cholecystitis is a rare but serious complication seen during prolonged hospital stays in patients receiving opiates. In our study, we evaluate methylnaltrexone’s effects and usage among trauma patients. Although this study has several important limitations, we did not see a statistically significant difference in the occurrence of cholestasis between those who received methylnaltrexone and those who did not.

## Conclusions

We present data from our level I trauma center comparing patients who received methylnaltrexone to those who did not. Our data suggests that patients who received the medication were overall sicker with longer hospital stays, increased mortality, and more likely to have required surgery. We did not show a significant difference in the incidence of cholestasis or acalculous cholecystitis between the two groups but suggest that a larger study is warranted to study a causal relationship.
